# Untargeted Metabolomics Reveals the Metabolic Characteristics and Biomarkers of Antioxidant Properties of Gardeniae Fructus from Different Geographical Origins in China

**DOI:** 10.3390/metabo15010038

**Published:** 2025-01-10

**Authors:** Wu Jiang, Lingling Jiang, Xiaoli Yin, Shuhui Zhang, Xiaojing Duan, Jiadong Chen, Yingying Liu, Hong Zheng, Zhengming Tao

**Affiliations:** 1 Zhejiang Institute of Subtropical Crops, Zhejiang Academy of Agricultural Sciences, Wenzhou 325005, China; jiangwu@zaas.ac.cn (W.J.); duanxj@zaas.ac.cn (X.D.); chenjd@zaas.ac.cn (J.C.); liuyy@zaas.ac.cn (Y.L.); 2 Innovation Center of Chinese Medicine Crops, Zhejiang Academy of Agricultural Sciences, Hangzhou 310021, China; 3 College of Science, Mathematics and Technology, Wenzhou-Kean University, Wenzhou 325060, China; jianglingling@wku.edu.cn; 4 Wenzhou Municipal Key Laboratory for Applied Biomedical and Biopharmaceutical Informatics, Wenzhou-Kean University, Wenzhou 325060, China; 5 School of Pharmaceutical Sciences, Wenzhou Medical University, Wenzhou 325035, China; yinxl@wmu.edu.cn (X.Y.); zhangsh@wmu.edu.cn (S.Z.); hongzheng@wmu.edu.cn (H.Z.)

**Keywords:** antioxidant, biomarker, metabolomics, flavonoids, geographical origin

## Abstract

**Background/Objectives:** Gardeniae Fructus (GF) has been widely used as both food and medicinal purposes for thousands of years, but their antioxidant properties and potential metabolite biomarkers remain unclear. **Methods**: The purposes of this study were to examine antioxidant activities of 21 GF varieties from different geographical origins in China and identify potential biomarkers of antioxidant properties using an untargeted LC–MS-based metabolomics approach. **Results**: The results demonstrate that metabolomics had the ability to trace the geographical origins of GF. We found that antioxidant activities varied with different varieties of GF, which was dependent on their chemical compositions. The key chemical categories were obtained as the primary contributors of the antioxidant activity, including prenol lipids, flavonoids, coumarins and derivatives, as well as steroids and steroid derivatives. In addition, adouetine Y, coagulin R 3-glucoside and epicatechin 3-glucoside were identified as potential biomarkers for the antioxidant activity of GF. **Conclusions**: Therefore, our study sheds light on the metabolic characteristics and biomarkers of the antioxidant properties of GF, contributing to the selection and cultivation of a high antioxidant variety.

## 1. Introduction

Gardeniae Fructus (GF) has been extensively utilized as the dried fruits of *Gardenia jasminoides* Ellis (family Rubiaceae) in traditional medicine across many Asian countries [1]. In China, the historical usage of GF can date back over 2000 years to the Han Dynasty, where it was recorded for use as both medicinal and dyeing agents [1]. Approximately 250 species of genus *Gardenia* are distributed across tropical and subtropical regions [2]. Among them, *Gardenia jasminoides* Ellis is widely cultivated in the provinces located at south of the Yangtze River in China, including Zhejiang, Jiangxi, Fujian, Hunan, Sichuan and Guizhou provinces [3]. Of note, GF has been recognized as a high-quality medicine and food homologous plant and attracted increasing interest due to its considerable commercial value. Yet, there are significant alterations in the chemical compositions of GF from different geographical origins, leading to confusion regarding the quality of GF in the market [4]. Hence, the clarification of the differences in chemical components of GF across various production areas is of great importance to consumer interests and for a healthy commercial market.

Recently, GF extracts are increasingly being incorporated into functional foods due to their main active constituents, such as iridoids, terpenoids, flavonoids, organic acids and lignans [5]. These components have been known to exert many health-promoting properties, including hepatoprotective, anti-inflammatory, antioxidant, neuroprotective and antitumor properties [5]. Oxidative stress has been regarded as a key factor of many diseases, so antioxidant therapy is becoming a promising method to prevent and treat diseases through dietary components and pharmaceutical intervention [6]. Therefore, there is great interest in examining the antioxidant properties of GF [7,8]. Antioxidant components, which can scavenge free radicals, are crucial for treating diseases by reducing oxidative stress and maintaining human health [9]. However, the changes in the chemical composition result in differences in antioxidant activities among different varieties [10,11] and geographical origins [12,13]. This highlights the significance of evaluating antioxidant activities and identifying potential biomarkers to ensure the high quality of functional foods. So far, some metabolite biomarkers of antioxidant properties have been identified in plant-based foods, including purple rice [14], grape seeds [15], sour cherry [16] and grains [17]. However, there is no information available on antioxidant activities and chemical biomarkers regarding the different geographical origins of GF.

In the present study, therefore, we analyzed the antioxidant activities of 21 varieties of GF from different regions of China by three evaluation systems and examined their metabolic characteristics via an untargeted LC–MS-based metabolomics method. The objectives of this study were to (i) examine the metabolic profiles and antioxidant properties of GF from different geographical origins, and (ii) identify the key chemical categories and biomarkers associated with antioxidant activities in GF.

## 2. Materials and Methods

### 2.1. Sample Collection and Preparation

In this study, 21 batches of Gardeniae Fructus (GF) were harvested from different geographical origins in China, including Zhejiang, Jiangxi, Sichuan, Chongqing, Hunan, Hubei, Fujian, and Guizhou provinces (Table 1). The plant samples were authenticated by Professor Zhengming Tao, an expert in plant taxonomy. Fresh fruits were rinsed with distilled water and dried at 50 °C until at a constant weight. The dried fruits were then finely ground using a stainless-steel grinder, passed through a 60-mesh sieve, and stored at room temperature in a dry condition until use.

### 2.2. Metabolite Extraction

GF was subjected to ultrasound-assisted extraction as described by Gong et al. [18] with minor modifications. Briefly, 0.2 g of the powder sample was weighed into an extraction tube, and mixed with 10 mL of distilled water and then sonicated (power 500 W) in an ultrasonic bath for 10 min. After extraction, the mixture was centrifuged at 5000× *g* at 4 °C for 5 min. The supernatant was transferred into a new tube and dried under nitrogen. The extract powder was stored at −80 °C until analysis.

### 2.3. Determination of Antioxidant Activity

The antioxidant properties of GF harvested from different geographical origins were assessed using three different approaches by detecting the scavenging percentages of DPPH, ABTS and superoxide radicals. The sample solution was prepared by redissolving 10 mg of the extract powder into 100 mL of distilled water. The DPPH and ABTS assays were conducted using the method described by Rumpf et al. [19] with minor modifications. For the DPPH assay, 0.1 mL sample solution was mixed with 3.9 mL DPPH radical solution and then kept in complete darkness for 30 min. The absorbance was measured at 517 nm. For the ABTS assay, 2.5 mL ABTS radical solution was mixed with 0.25 mL sample solution and the absorbance was determined at 734 nm after 30 min. Moreover, the superoxide radical scavenging activity was measured based on a previous method with minor modifications [20]. In brief, 0.35 mL of the sample solution was added to 0.75 mL of 2 mM NADH, 0.75 mL of 250 μM NBT solution and 0.37 mL of 5.4 μM PMS. The mixture was incubated for 30 min and the absorbance was measured at 560 nm. The radical scavenging activity (RSA) is calculated using the following formula:

(1) $$RSA\left(\%\right)=\left(1-\frac{R_{S}}{R_{C}}\right)*100$$

where *R_S_* and *R_C_* represent the absorbance of the sample and blank, respectively.

### 2.4. Untargeted LC–MS-Based Metabolomics Analysis

For LC–MS metabolomics analysis, 10 mg of the extract powder of GF was redissolved into 100 mL of acetonitrile/water (1:1, *v*/*v*) solution containing 0.02 mg/mL of L-2-chlorophenylalanine. Metabolite profiling of GF was performed using a Thermo UHPLC-Q Exactive system coupled with an ACQUITY BEH C18 column (100 × 2.1 mm, 1.7 µm, Waters, Milford, MA, USA). The chromatographic separation was achieved using a mobile phase consisting of 0.1% formic acid in water: acetonitrile (2:98, *v*/*v*) (solvent A) and 0.1% formic acid in acetonitrile (solvent B) with a flow rate of 0.4 mL/min. The solvent gradient was set as follows: (i) 0.5 min with 2% B, (ii) 0.5–7.5 min with a linear gradient from 2% to 35% B, (iii) 7.5–13 min with a linear gradient from 35% to 95% B, (iv) 13–14.4 min with 95% B, (v) 14.4–14.5 min with 95% to 2% B, and (vi) 14.5–16 min with 2% B. The injection volume was 3 μL and the column temperature was set at 40 °C. The mass spectrometer was equipped with an electrospray ionization (ESI) source, operating in both positive and negative modes. The main parameters were set as follows: source temperature, 400 °C; sheath gas flow rate, 40 arb; Aux gas flow rate, 10 arb; ion-spray voltage floating, −2800 V in negative mode and 3500 V in positive mode; normalized collision energy, 20-40-60 V rolling for MS/MS; mass range, 70–1050 *m*/*z*. Data acquisition was carried out with the data dependent acquisition (DDA) mode.

### 2.5. Data Analysis and Statistics

Raw LC/MS data were converted to mzXML format and preprocessed using Progenesis QI software (v2.3, Waters Corporation, Milford, MA, USA) for peak alignment, peak picking, peak integration and retention time (RT) correction. Then a three-dimensional data matrix containing the *m*/*z* value, RT and peak intensity was exported in CSV format. The peak intensities detected at least 80% in any set of samples were retained and normalized by sum. Meanwhile, variables with relative standard deviation (RSD) greater than 30% of quality control samples were removed and log 10 transformation was applied to obtain the final data matrix for further analysis. In this study, metabolites were simultaneously identified using the MJDBPM database, developed by Shanghai Majorbio Bio-Pharm Biotechnology Co., Ltd. (Shanghai, China).

Principal component analysis (PCA) was used to examine the differences in metabolic patterns of GF from different geographical origins by SIMCA-P+ software (v12.0, Umetrics AB, Umea, Sweden). Orthogonal projection to latent structures discriminant analysis (OPLS–DA) was utilized to obtain metabolic differences between two groups and identify important contributive metabolites by S-plot. Partial least squares regression (PLSR) model was conducted to analyze the relationship between antioxidant activities and metabolites and identify key metabolites by the variable importance in the projection (VIP) method using SIMCA-P+ software (v12.0), and metabolites with a VIP value > 1.0 were selected for further analysis. The cluster analysis was conducted using the SciPy package in Python (Version 1.0.0), applying Euclidean distance with the Ward method. Enrichment analyses based on the KEGG pathways and chemical structures were performed using the MetaboAnalyst 5.0 [21]. Linear correlation analysis was carried out to evaluate the relationship between the average antioxidant activities and metabolites by Microsoft office excel 2007. The statistic difference among different groups was analyzed by ANOVA using SPSS 22.0 software (SPSS, Inc., Chicago, IL, USA) and statistical significance was defined at *p* < 0.05.

## 3. Results and Discussion

### 3.1. Tracing the Geographical Origins of Gardeniae Fructus by Metabolomics

**Figure 1:**
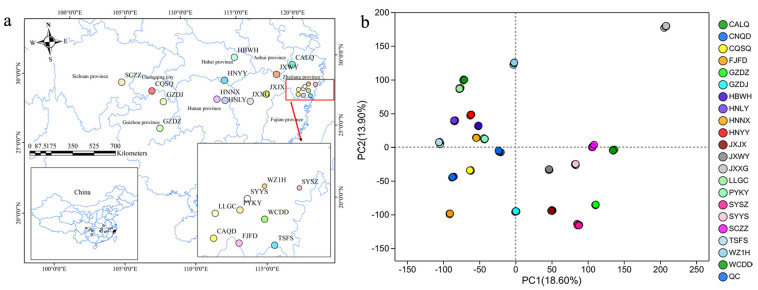
Tracing the geographical origins of Gardeniae Fructus by using an untargeted LC–MS-based metabolomics method. (**a**) The geographical distribution of 21 varieties of GF; (**b**) PCA classification of 21 GF varieties based on metabolomics data.

To examine the metabolic characteristics of Gardeniae Fructus (GF), we collected 21 varieties distributed across different regions of China for untargeted LC–MS-based metabolomics analysis (Figure 1a). The PCA results (Figure 1b) demonstrate that there were clear separations in the metabolic patterns among different varieties of GF, suggesting that metabolomics has the ability to discriminate between the geographical origins of GF. Li et al. [22] also used an LC–MS-based metabolomics method to distinguish the source of *Lycium barbarum* L. (LB) and identify 21 metabolites that exhibited high sensitivity and specificity as potential biomarkers of LB. Lushan Yunwu teas (LYTs) from 8 different geographical origins were differentiated by untargeted metabolomics, and altitude was considered as the main influencing factor for the chemical compositions of LYTs [23]. Using LC–MS-based metabolomics, region-specific metabolites were identified to discriminate *Amomum villosum* fruits (AVF) from 5 regions by Li et al. [24], who reported 11 environmental factors that affect the metabolites of AVF. Moreover, metabolomics has also been employed to differentiate the geographical origins of foods or drugs from various sources, including plant [25,26,27], animal [28,29,30] and microorganism [31]. Therefore, it can be seen that metabolomics is a promising tool to distinguish the geographical origins of GF.

### 3.2. Chemical Composition of Gardeniae Fructus

**Figure 2:**
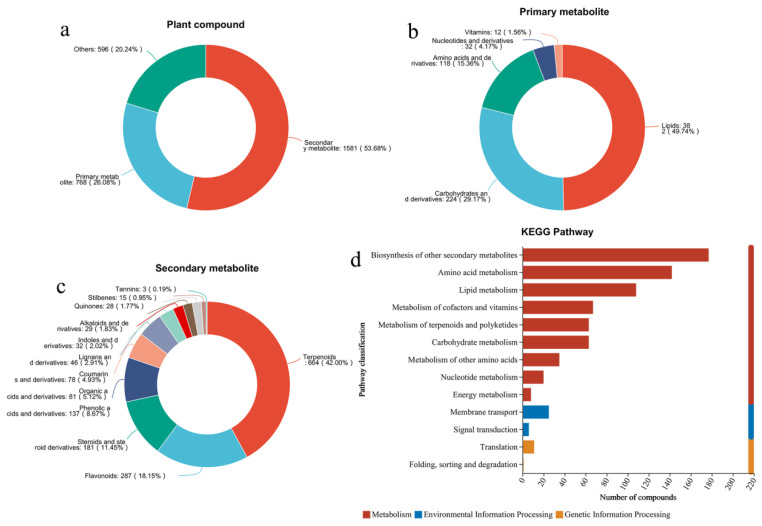
Chemical composition of Gardeniae Fructus. (**a**) Plant compound; (**b**) Primary metabolite; (**c**) Secondary metabolite; (**d**) Metabolic pathway analysis based on KEGG database.

Figure 2 illustrates the chemical composition and metabolic pathways of GF. We detected that the plant compounds in GF mainly consisted of 26.08% primary metabolites and 53.68% secondary metabolites (Figure 2a). In primary metabolites, the predominant component consisted of lipids (49.74%), followed by carbohydrates and derivatives (29.17%), amino acids and derivatives (15.36%), nucleotides and derivatives (4.17%), and vitamins (1.56%), as shown in Figure 2b. The secondary metabolites are primarily composed of 42.00% terpenoids, 18.15% flavonoids, 11.45% steroids and steroid derivatives, 8.67% phenolic acids and derivatives, 5.12% organic acids and derivatives, and others (Figure 2c). These components have been reported to exhibit antioxidant activity [32,33]. In addition, the metabolic pathway analysis shows key metabolic pathways in GF, including mainly biosynthesis of other secondary metabolites, amino acid metabolism, lipid metabolism, metabolism of cofactors and vitamins, metabolism of terpenoids and polyketides, carbohydrate metabolism, and others (Figure 2d).

**Figure 3:**
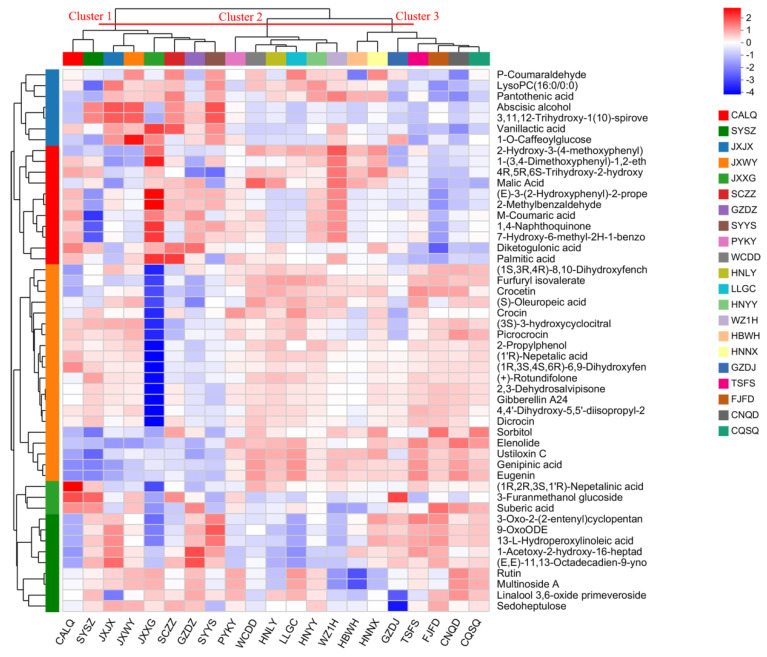
Metabolic changes in Gardeniae Fructus. Cluster analysis based on top 50 significantly differentiated metabolites among different varieties.

Furthermore, the cluster analysis reveals that GF can be clustered into three groups based on top 50 significantly differentiated metabolites, as illustrated in Figure 3. Cluster 1 included CALQ, SYSZ, JXJX, JXWY, JXXG, SCZZ, GZDZ and SYYS, and was characterized by lower levels of sorbitol, elenolide, ustiloxin C, genipinic acid and eugenin. Cluster 2 comprised the following varieties: PYKY, WCDD, HNLY, LLGC, HNYY and WZ1H. Figure 3 shows that the primary feature of the cluster 2 was lower levels of vanillactic acid, 1-O-caffeoylglucose, suberic acid, 9-OxoODE, 13-L-hydroperoxylinoleic acid, and rutin. Cluster 3 consisted of HBWH, HNNX, GZDJ, TSFS, FJFD, CNQD and CQSQ and contained higher levels of furfuryl isovalerate, crocetin, (S)-oleuropeic acid, crocin, picrocrocin, 2-propylphenol, (1’R)-nepetalic acid, (+)-rotundifolone, 2,3-dehydrosalvipisone, gibberellin A24, dicrocin, sorbitol, elenolide, ustiloxin C, genipinic acid, eugenin, 9-OxoODE, 13-L-hydroperoxylinoleic acid, and rutin (Figure 3). Thus, it is evident that different varieties of GF contained varying levels of chemical components, which in turn influences their bioactive functions, such as their antioxidant and anti-inflammatory activities.

### 3.3. Antioxidant Activity of Gardeniae Fructus

**Figure 4:**
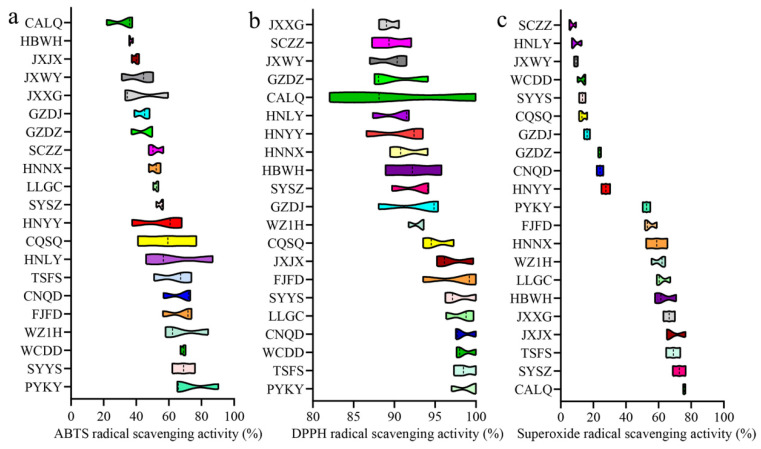
Antioxidant activity of Gardeniae Fructus. Antioxidant activities were evaluated by calculating (**a**) ABTS, (**b**) DPPH and (**c**) superoxide radical scavenging activities. Different colors represent different Gardenia Fructus varieties.

To examine antioxidant activities in different varieties of GF, we utilized three different evaluation systems: ABTS, DPPH and superoxide assays. The results show that the average ABTS and superoxide radical scavenging activities of GF ranged from 31.81% to 73.75% (Figure 4a) and from 6.76% to 75.78% (Figure 4c), respectively. However, all GF studied herein had an average DPPH radical scavenging activity above 89.24% (Figure 4b). Of note, PYKY exhibited the highest antioxidant activities in the ABTS (73.75%) and DPPH (99.00%) assays, but not in the superoxide assay (52.65%). For superoxide radical scavenging activity, CALQ was the most prominent, with an average antioxidant activity of 75.78% (Figure 4c). In addition, there were three varieties of GF, including TSFS, FJFD and WZ1H, which consistently rank among the top ten for antioxidant activity (Figure 4). These results imply that GF had varying antioxidant activities under different evaluation systems. It is well known that antioxidants play a crucial role in human health, such as in anti-inflammation [34], anti-obesity [35], antidiabetic [36] and anticancer [37]. Therefore, the evaluation of antioxidant activity will aid in selecting more suitable varieties of GF to achieve better health-promoting effects.

### 3.4. Analysis of Main Chemical Compositions Contributable to Antioxidant Activity

To investigate the relationship between chemical compounds and antioxidant ability, we predicted the antioxidant activity of GF by a PLSR model based on metabolomics data. We found that there were good correlations between predicted and experimental values in ABTS (R^2^ = 0.8136), DPPH (R^2^ = 0.7046) and superoxide (R^2^ = 0.7246) assays, suggesting that the antioxidant capacity of GF is determined by their chemical composition.

**Figure 5:**
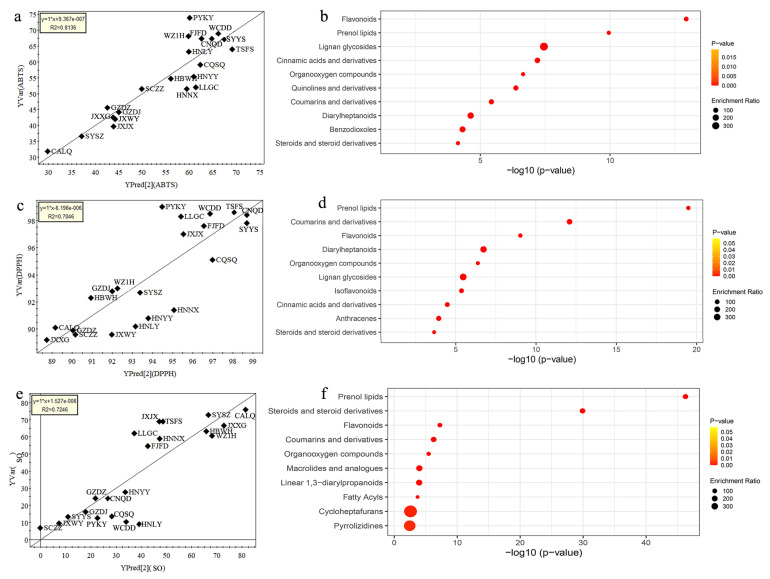
Analysis of main chemical compositions contributable to antioxidant activity. PLSR models showing the correlations between predicted and experimental values in (**a**,**b**) ABTS, (**c**,**d**) DPPH and (**e**,**f**) superoxide assays, and the corresponding enrichment analyses based on chemical structures showing primary categories of antioxidant compounds in Gardeniae Fructus.

Subsequently, the enrichment analysis based on chemical structures of important metabolites identified from PLSR models (VIP > 1.0) was performed, and the top ten chemical categories contributing mainlyu to antioxidant activity of GF are presented in Figure 5b for ABTS assay, Figure 5d for DPPH assay and Figure 5f for superoxide assay. Of note, prenol lipids were identified as a common significant chemical category for the antioxidant activity of GF in all three evaluation systems (Figure 5). Prenol lipids belong to plant secondary metabolites synthesized from the five carbon precursors [38] and enable plants to resist environmental stress [39,40]. Moreover, these lipids also have antioxidant effects, including carotenoids [41], poly-terpenes [42], and vitamin E [43]. For ABTS scavenging activity, flavonoids were identified as the primary contributors (Figure 5b). Flavonoids as secondary metabolites are a group of polyphenolic substances and exist widely in plant-based foods or drugs [44]. Flavonoids have been proved to have high antioxidant effects and protect against multiple diseases, including colitis, obesity, diabetes, cancer, and others [44,45]. We found that coumarins and derivatives mainly contributed to DPPH scavenging activity of GF (Figure 5d). Coumarins have been regarded as natural antioxidants [46,47] and possess broad pharmacological value [48]. Steroids are a unique group of chemical compounds existed in both plants and animals [49]. In this study, steroids and steroid derivatives was identified to be mainly responsible for superoxide scavenging activity (Figure 5f). Antioxidant properties of steroids have been found in animals including estriol and 17β-estradiol [50]. Plant steroids have many important medicinal and pharmaceutical properties beyond their role as a plant growth regulator, such as anticancer, antibacterial and anti-inflammatory activity [49,51,52]. Moreover, some new plant steroids have been discovered as potential antioxidant agents [53,54]. Taken together, we have revealed key categories of antioxidant compounds in GF, which will facilitate the selection and cultivation of superior GF varieties.

### 3.5. Biomarkers of High Antioxidant Property in Gardeniae Fructus

**Figure 6:**
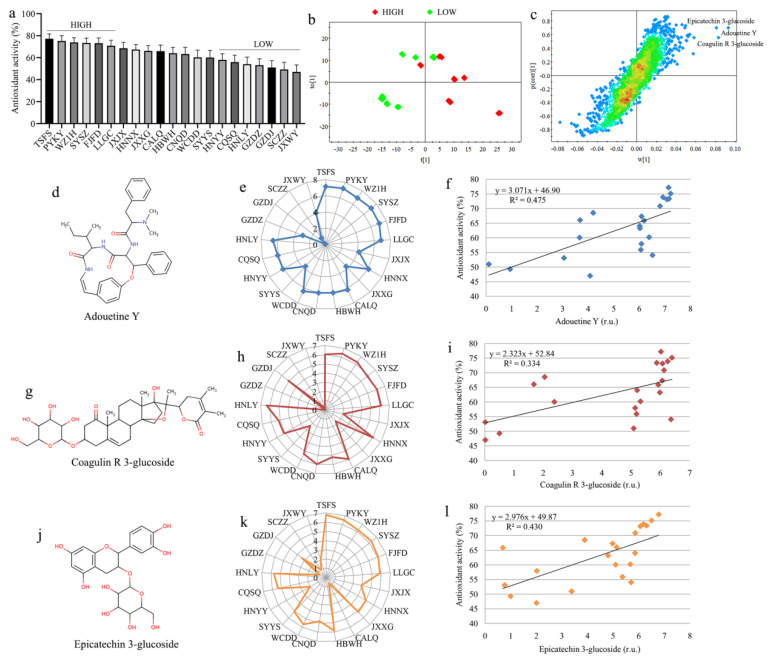
Biomarkers of high antioxidant property in Gardeniae Fructus. (**a**) Average antioxidant activities (AAA) in different varieties of GF, and defined GF with AAA values > 70% and <50% were defined as high antioxidant varieties (HIGH) and low antioxidant varieties (LOW), respectively; (**b**) OPLS-DA model between the HIGH and LOW groups based on metabolomics data and (**c**) the corresponding S-plot. Blue, green, yellow and red represent the plot density from low to high.; Molecular structures of (**d**) adouetine Y, (**g**) coagulin R 3-glucoside and (**j**) epicatechin 3-glucoside; Changes in the levels of (**e**) adouetine Y, (**h**) coagulin R 3-glucoside and (**k**) epicatechin 3-glucoside in different varieties of GF; Correlations of AAA with (**f**) adouetine Y, (**i**) coagulin R 3-glucoside and (**l**) epicatechin 3-glucoside.

Herein, we calculated the average antioxidant activity (AAA) and defined GF with an AAA value greater than 70% as high antioxidant varieties (HIGH), while those with an AAA value less than 50% were classified as low antioxidant varieties (LOW), as shown in Figure 6a. The HIGH group consisted of six varieties including TSFS, PYKY, WZ1H, SYSZ, FJFD and LLGC, and we found that these HIGH varieties are cultivated in Wenzhou, Zhejiang, and its surrounding areas. This suggests that GF from the Wenzhou region exhibited a notably higher antioxidant activity.

Then, an OPLS–DA model was used to examine the metabolic difference between the HIGH and LOW groups (Figure 6b) and identify key metabolites via S-plot (Figure 6c). The results show that the metabolic pattern of the HIGH group was clearly different from that of the LOW group, and adouetine Y (Figure 6d), coagulin R 3-glucoside (Figure 6g) and epicatechin 3-glucoside (Figure 6j) were identified as important metabolites that mainly contributed to the metabolic separation between two groups in Figure 6b. Relative to other varieties, GF in the HIGH group had higher levels of adouetine Y (Figure 6e), coagulin R 3-glucoside (Figure 6h) and epicatechin 3-glucoside (Figure 6k). In addition, we found that the AAA values of GF were positively correlated with adouetine Y (R^2^ = 0.475, Figure 6f), coagulin R 3-glucoside (R^2^ = 0.334, Figure 6i) and epicatechin 3-glucoside (R^2^ = 0.430, Figure 6l). Adouetine Y is an alkaloid that has shown potential as an antioxidant [55]. Yamin et al. [56] also reported its potential antiviral activity particularly for SARS-CoV-2. Coagulin R 3-glucoside is a compound belonging to the class of steroids and steroid derivatives, and also exerts free radical scavenging activity [57]. Additionally, epicatechin 3-glucoside is one of the flavonoid compounds that have been widely recognized as natural antioxidants [44,58]. Collectively, our results indicate that these three metabolites might be utilized as potential biomarkers for the antioxidant activity of GF.

## 4. Conclusions

In this study, we reported that the geographical origins of GF can be distinguished via an LC–MS-based metabolomics. The antioxidant capacity varied among different varieties of GF, depending on the variations in their chemical components. Additionally, three metabolites, i.e., adouetine Y, coagulin R 3-glucoside and epicatechin 3-glucoside, were identified as potential biomarkers of the antioxidant activity of GF. Our study not only characterizes the metabolomic profiles and antioxidant activity of different varieties of GF, but also offers a reference for the selection and cultivation of GF with high antioxidant activity.

## Figures and Tables

**Table 1:** Detailed information on GF in this study.

Sample No.	Origins	Longitude (E)	Latitude (N)	Altitude (m)
JXXG	Xingan, Jiangxi	115°18′47.47″	27°44′45.01″	122
JXWY	Wuyuan, Jiangxi	117°46′51.87″	29°25′52.55″	126
JXJX	Jinxi, Jiangxi	116°42′39.07″	28°05′49.09″	159
HNYY	Yueyang, Hunan	113°22′48.02″	29°30′01.02″	660
HNNX	Ningxiang, Hunan	112°36′36.04″	28°10′47.99″	920
HNLY	Liuyang, Hunan	113°15′30.24″	28°02′07.42″	76
HBWH	Wuhan, Hubei	114°26′48.83″	31°06′46.89″	110
GZDJ	Dejiang, Guizhou	108°10′08.65″	28°15′41.22″	710
GZDZ	Danzhai, Guizhou	107°48′42.63″	26°19′57.55″	828
FJFD	Fuding, Fujian	120°15′11.88″	27°25′39.31″	295
CQSQ	Sanquan, Chongqing	107°15′36.03″	29°05′23.98″	1050
SCZZ	Zizhong, Sichuan	104°43′36.89″	29°45′09.91″	437
CALQ	Chunan, Zhejiang	119°14′02.77″	29°56′12.87″	445
CNQD	Cangnan, Zhejiang	120°17′41.43″	27°24′32.66″	312
PYKY	Pingyang, Zhejiang	120°29′46.33″	27°41′01936″	404
TSFS	Taishun, Zhejiang	120°14′28.01″	27°26′24.97″	430
WCDD	Wencheng, Zhejiang	120°09′37.86″	27°50′28.60″	508
SYYS	Yongjia, Zhejiang	120°33′59.70″	28°10′14.08″	101
LLGC	Yongjia, Zhejiang	120°32′24.04″	28°13′12.04″	280
SYSZ	Yongjia, Zhejiang	120°35′24.06″	28°17′24.34″	460
WZ1H	Yongjia, Zhejiang	120°32′19.18″	28°17′19.18″	408

## Data Availability

Dataset available on request from the authors.
